# Annual Meeting of the Dental Protective Association

**Published:** 1891-03

**Authors:** 


### ANNUAL MEETING OF THE DENTAL PROTECTIVE ASSOCIATION.

THE report of the annual meeting of this Association will be found on another page. This is satisfactory, showing that the profession is generally being aroused to the importance of the subject. The amount of energy that Dr. Crouse has devoted to this should stimulate to corresponding efforts on the part of those equally interested.

It will be observed that the statement is made that they have "driven the Tooth Crown Company from Milwaukee, and that a suit has been commenced in New York, and that answers will be filed in time." The report also claims that the Association has saved one million dollars to the dental profession in the past year.

Notwithstanding the very extended notices issued of the aims and objects of this Association, there is still a want of definite legal knowledge on the part of dentists in regard to it, and as a consequence much bad feeling has been engendered. Whether this is the fault of individuals or of the Protective Association it is difficult to determine; but the fact remains that many are still in doubt, especially those who have heretofore worked under a license. Some means should be taken to clear up the latter question.

